# When Pain Shapes Dental Anxiety: A Cross-Sectional Mediation Study in Patients Requiring Endodontic Treatment

**DOI:** 10.3390/jcm15124660

**Published:** 2026-06-16

**Authors:** Edanur Maraş, Özge Başar, İlyas Çolakoğlu

**Affiliations:** 1 Department of Endodontics, Faculty of Dentistry, Recep Tayyip Erdoğan University, Rize 53100, Turkey; edanur.maras@erdogan.edu.tr; 2 Department of Endodontics, Faculty of Dentistry, Adnan Menderes University, Aydın 09010, Turkey; 3 Faculty of Dentistry, Recep Tayyip Erdoğan University, Rize 53100, Turkey; ilyas_colakoglu21@erdogan.edu.tr

**Keywords:** endodontics, dental anxiety, fear, pain, mediation analysis

## Abstract

**Objective**: This study aimed to evaluate the associations between dental anxiety–fear, state–trait anxiety, and pain in patients requiring endodontic treatment and to explore the role of preoperative pain intensity in these associations. **Methods**: This cross-sectional study included 253 adults scheduled for endodontic treatment. Sociodemographic characteristics and relevant clinical data were recorded. Pain intensity and psychological variables were assessed using the Numerical Rating Scale (NRS), Modified Dental Anxiety Scale (MDAS), Dental Fear Survey (DFS), and the State Anxiety and Trait Anxiety subscales of the State–Trait Anxiety Inventory (STAI-S and STAI-T). Associations were analyzed using group comparison tests and correlation analyses, and mediation analyses were performed using Hayes’ PROCESS macro-4. **Results**: Significant correlations were found among all psychological parameters (*p* < 0.05). The observed associations were consistent with a potential partial mediating role of pain in the relationship between state–trait anxiety and dental anxiety. Higher anxiety levels were present in patients with anterior teeth requiring either primary treatment or retreatment compared with posterior teeth (*p* < 0.05). **Conclusions**: Preoperative pain may contribute to the association patterns identified between dental anxiety and state–trait anxiety. Further studies with larger sample sizes and adjusted analytical models are needed to better clarify these relationships.

## 1. Introduction

Endodontic treatment plays an important role in preserving natural teeth and supporting subsequent restorative or prosthetic treatment. Complaints related to the dental pulp and periapical tissues are among the most common reasons for seeking dental care [1]. However, dental care is also commonly associated with pain, fear, and anxiety, which may cause patients to delay or avoid care [2]. Although microbial and biological aspects of pulpal and periapical disease have been extensively investigated, the psychological dimensions of the endodontic pain experience have received comparatively less attention [3]. Emotional responses, pain expectations, and anxiety may influence treatment perception, procedural tolerance, and compliance with future dental care. Accordingly, understanding these psychological factors before endodontic treatment may improve patient management and treatment planning [4].

Dental fear refers to an emotional or behavioral response to a specific and identifiable threat [5]. Dental anxiety, in contrast, is a more generalized emotional state arising from uncertainty, loss of control, and expectations regarding treatment [6]. Although these concepts are often used interchangeably, both are influenced by internal factors, such as personality traits, and external factors, including previous negative dental experiences and different sources of information [5,6].They often develop during childhood and have been reported to affect 10–29% of adults [5,7]. Dental anxiety–fear may cause patients to avoid dental appointments and display negative behavior during treatment/follow-up appointments [6].This avoidance cycle may worsen oral health and persist until pain or severe symptoms ultimately force individuals to seek treatment [2,8]. Such delays may complicate subsequent dental care and treatment planning [9].

Tooth pain is one of the most common types of orofacial pain and a major reason for seeking dental care [10]. The association between pain perception and psychological factors highlights the role of anxiety in shaping patient responses prior to endodontic treatment. Anxiety is commonly classified as state anxiety, a temporary response to a specific situation, and trait anxiety, a stable tendency to experience anxiety across different contexts [11,12,13,14]. Previous studies have reported correlations between dental anxiety, trait anxiety, and pain perception [11,12,14]. Individuals with high anxiety levels tend to expect greater pain during dental procedures and report higher pain intensity [12,14].

In the literature, dental anxiety–fear, pain, and state–trait anxiety have largely been investigated separately, with most studies focusing on expected rather than preoperative pain [3,11,15,16,17]. Furthermore, although pain and anxiety are known to be associated, the potential mediating role of pain in the relationship between dental anxiety–fear and state–trait anxiety has not been previously investigated [12,14,18]. Therefore, this study aimed to investigate the correlations between preoperative pain, dental anxiety–fear, and state–trait anxiety and to evaluate whether the observed associations were consistent with an indirect role of pain. The null hypotheses are as follows: (i) there are no correlations between these variables, (ii) pain does not mediate the relationship between dental anxiety–fear and state–trait anxiety, and (iii) there are no differences in these measures according to demographic, behavioral, and endodontic characteristics.

## 2. Materials and Methods

This study was conducted in full accordance with the applicable ethical principles, including the World Medical Association Declaration of Helsinki. The study protocol was approved by the Research Ethics Committee of Recep Tayyip Erdoğan University (RTEU; approval number 2024/196, date: 18 July 2024). Patient recruitment was initiated following ethical approval. The study included patients who presented to the Department of Endodontics, Faculty of Dentistry, RTEU. All data were collected before the clinical procedure, and written informed consent was obtained from all participants. The minimum required sample size was calculated as 200 with G*Power software (ver. 3.1.9.7; Heinrich Heine University Düsseldorf, Düsseldorf, Germany), assuming a statistical power of 0.80, an effect size of 0.2, and *α* = 0.05 [19]. A total of 253 participants were enrolled during the data collection period. The post hoc power for this final sample size was calculated as 89%. The inclusion criteria comprised adults aged 18–65 years, classified as ASA I–III, with sufficient Turkish language proficiency and the ability to provide informed consent and complete the questionnaires. Participants were excluded if they had any significant medical or psychiatric conditions, communication difficulties, substance abuse, malignancy, pregnancy, or breastfeeding; if they declined participation; or if they were using medications that could influence anxiety, including selective serotonin reuptake inhibitors. In addition, individuals who had used medications capable of altering pain perception (e.g., analgesics or anti-inflammatory drugs) within the preceding 12 h prior to treatment, or who had received systemic antibiotic therapy within the last month before the study, were excluded.

### 2.1. Patient Interview and Data Collection

Sociodemographic and clinical variables were recorded for each participant, including age, sex, educational level, employment status, monthly income category, frequency of dental visits, history of traumatic dental experiences, primary source of dental information, waiting time before treatment, prior knowledge about the planned treatment, and history of previous endodontic treatment.

### 2.2. Endodontic Examination

For each patient, the numbers of decayed (D), missing (M), and filled (F) teeth were recorded to calculate the Decayed, Missing, and Filled Teeth (DMFT) index. The presence of removable dental prostheses was also recorded. The evaluation included identifying teeth requiring primary or secondary endodontic therapy, teeth with previous root canal treatment, and the presence of periradicular radiolucency. If multiple teeth required endodontic treatment, the symptomatic tooth was selected for diagnostic classification, and its periapical radiolucency was assessed using the Periapical Index (PAI) classification described by Ørstavik et al. [20]. For multirooted teeth, the highest score among the roots was recorded.

### 2.3. Questionnaires, Scales, and Physical Parameters

Dental Anxiety: Dental anxiety levels were assessed using the Modified Dental Anxiety Scale (MDAS), developed by Humphris et al. [21]. This self-report instrument consists of five items, each rated on a 5-point scale ranging from 1 (no anxiety) to 5 (extreme anxiety), yielding a maximum total score of 25. The validated Turkish version of the MDAS was used in this study [22].

Dental Fear: Dental fear was evaluated using the Dental Fear Survey (DFS), a widely used instrument with established psychometric properties [23]. The DFS consists of 20 items, each rated on a five-point scale ranging from 1 (low) to 5 (high). In the present study, the version previously validated and used by Kvale et al. was applied [24].

Pain Assessment: Preoperative pain intensity was assessed using the Numerical Rating Scale (NRS), a simple but sensitive measure validated across various clinical settings [25]. This unidimensional 11-point scale (0 = no pain, 10 = unbearable pain) can be administered verbally or in written form. Pain scores were categorized as 0, indicating no pain; 1–3, mild pain; 4–6, moderate pain; and 7–10, severe pain.

Anxiety Assessment: Levels of state and trait anxiety were measured using the State–Trait Anxiety Inventory (STAI). In the present study, the Turkish version of the STAI was used [26]. The State Anxiety subscale of the State–Trait Anxiety Inventory (STAI-S) and the Trait Anxiety subscale of the State–Trait Anxiety Inventory (STAI-T) were administered separately. Each subscale consists of 20 items scored on a four-point Likert scale. Negative statements are scored directly, whereas positive statements are reverse scored. Higher total scores indicate higher levels of anxiety.

The MDAS, STAI-T, and STAI-S questionnaires and scales were administered in a separate, standardized, and quiet clinical area containing a single examination unit to minimize the influence of treatment-related stimuli and interactions with other patients. The questionnaires and scales were completed immediately before treatment under the supervision of the researchers (EM and IC).

Pulse rate and oxygen saturation were measured by the researcher (EM) using a fingertip pulse oximeter (Resiprox; Contec Medical, Qinhuangdao, China) under standardized conditions.

### 2.4. Interobserver Reliability Analysis

Endodontic evaluations were performed by an endodontist with more than 8 years of clinical experience (EM). To assess interobserver reliability, the presence of caries and PAI scores were independently evaluated by EM and a second endodontist (OB) with more than 5 years of clinical experience. The intraclass correlation coefficient (ICC) for caries assessment (0.996) and the weighted Cohen’s kappa coefficient for PAI (0.955) indicated excellent interobserver agreement for both measurements [27,28].

### 2.5. Statistical Analysis

Descriptive statistics were calculated for all variables. Normality was assessed using the Shapiro–Wilk test and homogeneity of variances using Levene’s test. Depending on distributional assumptions, group comparisons were performed using the independent samples *t*-test or Mann–Whitney *U* test for two groups and one-way ANOVA or the Kruskal–Wallis test for three or more groups. Post hoc tests (Bonferroni or adjusted Bonferroni) were applied to determine which specific groups differed. Spearman correlation was used to evaluate associations between continuous variables, whereas Kendall’s Tau was applied for relationships involving ordinal measures. Mediation analyses were performed using Hayes’ PROCESS macro-4 (Andrew F. Hayes, Columbus, OH, USA). All analyses were conducted using IBM SPSS Statistics version 27 (IBM Corp., Armonk, NY, USA).

## 3. Results

### 3.1. Demographic, Clinical, and Endodontic Characteristics of the Participants

The demographic and clinical characteristics of the participants are presented in Table 1. Most participants were female and unemployed, and the majority reported visiting the dentist only when experiencing symptoms.

In terms of oral health status, caries (73.1%), missing teeth (69.6%), and the presence of restorations (81.8%) were common. Previous root canal treatment was reported in 65.6% of cases, and most teeth requiring endodontic treatment were located in the posterior region (primary treatment, 83.2%; retreatment, 77.1%) (Supplementary Table S1).

### 3.2. Reliability of the Study Scales

Internal consistency was high for the MDAS (*α* = 0.881), STAI-S (*α* = 0.870), and STAI-T (*α* = 0.831), and excellent for the DFS (*α* = 0.973) (Supplementary Table S2).

### 3.3. Associations Among Pain, Dental Anxiety, Dental Fear, and State–Trait Anxiety Scales

All psychological parameters and pain intensity were significantly correlated. Moderate positive correlations were observed between MDAS and NRS, MDAS and DFS, DFS and STAI-T, and STAI-S and STAI-T (*p* < 0.05). The remaining correlations involving NRS and anxiety measures were weak but statistically significant (*p* < 0.05; Table 2).

### 3.4. Mediation Analysis of the Associations Between State–Trait Anxiety, Pain, and Dental Anxiety–Fear

The observed indirect association patterns were consistent with the possibility that preoperative pain may contribute to the relationships between state anxiety (*β* = 0.0294; 95% CI: 0.0100–0.0554) and dental anxiety, as well as between trait anxiety (*β* = 0.0289; 95% CI: 0.0066–0.0568) and dental anxiety (Table 3).

No statistically significant indirect association patterns involving preoperative pain were found in the relationships between state or trait anxiety and dental fear, as the 95% confidence intervals included zero (Supplementary Tables S3 and S4).

### 3.5. Pain, Dental Anxiety–Fear, and State–Trait Anxiety Across Demographic and Behavioral Characteristics

Female and unemployed participants had significantly higher scores for the MDAS and DFS (*p* < 0.05). Participants who lacked prior information about the procedure exhibited significantly higher levels of dental anxiety–fear and state–trait anxiety compared with informed participants (*p* < 0.05). Furthermore, participants with higher income reported higher NRS and MDAS scores than those with lower income (*p* < 0.05; Table 4).

### 3.6. Pain, Dental Anxiety–Fear, and State–Trait Anxiety Across Endodontic Variables

In analyses based on endodontic variables, both state and trait anxiety scores were significantly higher in patients with previously treated anterior teeth than in those with previously treated posterior teeth (*p* < 0.05). Similarly, among patients requiring retreatment, state anxiety scores were significantly higher when the affected tooth was located in the anterior rather than posterior region (*p* < 0.05; Table 5).

### 3.7. Associations Between Demographic and Endodontic Characteristics and Pain, Dental Anxiety–Fear, and Anxiety Scores

Weak but significant negative correlations were noted between NRS pain intensity and age, number of missing teeth, and DMFT score, as well as between STAI-S and STAI-T scores and oxygen saturation. A weak but significant positive correlation was also observed between pulse rate and NRS pain intensity (*p* < 0.05; Supplementary Table S5).

## 4. Discussion

Pain and anxiety are important components of the patient experience during endodontic treatment and are influenced by both biological and psychological factors [29]. This study investigated the correlations between preoperative pain, dental anxiety–fear and state–trait anxiety in patients requiring endodontic treatment and aimed to assess whether the observed associations were consistent with a potential mediating role of pain.

In the present study, significant correlations were found between dental anxiety–fear, state–trait anxiety, and pain intensity, leading to the rejection of the first null hypothesis. Similarly, Dou et al. [4] reported a significant association between preoperative pain and dental anxiety, while Quartana et al. [30] and Murillo-Benítez et al. [31] reported that higher anxiety levels were associated with increased pain perception and pain-related responses during dental treatment. In contrast to the generally consistent associations reported in the literature, Fuentes et al. [11] proposed that dental anxiety was largely independent of trait anxiety. These differences may be related to methodological variations between studies. While Fuentes et al. evaluated a large general-population sample using Corah’s Dental Anxiety Scale (DAS), the present study specifically included patients requiring endodontic treatment and assessed dental anxiety using the MDAS. Taken together, these findings suggest that preoperative pain and anxiety-related variables are associated with dental anxiety in patients undergoing endodontic treatment, although the associations were generally small to moderate in magnitude and should therefore be interpreted cautiously [17].

A key finding of the present study was that the observed associations were consistent with a potential mediating role of preoperative pain in the relationship between state–trait anxiety and dental anxiety Although previous studies have reported associations among general anxiety, dental anxiety, and pain [11,32,33,34], to the best of our knowledge, no previous study has specifically explored whether indirect association patterns involving preoperative pain may contribute to these relationships. Therefore, direct comparisons with the present findings are limited.

Although the underlying mechanisms were not investigated in the present study, previous research has suggested that increased threat perception and attentional focus on pain-related stimuli may contribute to the co-occurrence of anxiety and heightened pain perception [35,36]. Accordingly, these explanations should be regarded as possible hypotheses rather than conclusions derived from the current findings [36]. The effect of state anxiety on pain intensity appeared to be more pronounced than that of trait anxiety. Notably, the observed indirect effects were modest in magnitude. Accordingly, the findings should be interpreted as reflecting indirect association patterns rather than evidence of a causal mediating mechanism, because the cross-sectional design does not permit conclusions regarding temporal or causal relationships among anxiety, pain, and dental anxiety. From a clinical perspective, these associations may indicate that anxiety and pain are related factors that could warrant consideration during patient management. Approaches such as clear communication, positive reinforcement, cognitive restructuring, and coping-oriented strategies have been proposed to improve patients’ treatment experiences [36,37]. However, the effectiveness of such approaches and their relevance to the relationships observed in the present study require confirmation through prospective and interventional research [32,33,36,37].

Unlike dental anxiety, no indirect association patterns involving pain were observed in the relationships between state–trait anxiety and dental fear. This finding may support the theoretical distinction between dental anxiety and dental fear. Although these concepts are frequently used interchangeably, they represent different psychological constructs. Previous research suggests that dental fear is more strongly linked to prior negative experiences and conditioned responses, whereas dental anxiety may show stronger associations with current pain experiences and treatment-related expectations [37]. Therefore, the present findings may suggest that pain is more closely associated with dental anxiety than with dental fear, although these observations should be interpreted cautiously given the cross-sectional nature of this study [37,38].

Regarding demographic and behavioral characteristics, the majority of patients reported visiting the dentist only after symptoms had developed. This pattern may be consistent with associations previously described in the literature between avoidance behavior, delayed treatment seeking, higher pain levels, and greater dental anxiety [2]. The high prevalence of caries, missing teeth, and restorations may reflect oral health conditions associated with delayed dental attendance [39]. Additionally, the high unemployment rate and educational profile of the sample may reflect socioeconomic factors associated with reduced access to preventive dental care and poorer oral health outcomes. Moreover, the higher dental anxiety–fear and state–trait anxiety levels observed among patients who had not been informed about the treatment procedure may highlight the potential importance of perceived control and preoperative communication. Previous studies have shown that clear and understandable explanations may strengthen patients’ sense of control and reduce dental anxiety [3,40]. Accordingly, assessment of pain and anxiety-related characteristics may provide additional information that could assist clinicians in identifying patients who may benefit from tailored communication [41].

Higher dental anxiety–fear in women is consistent with previous studies suggesting that women may perceive threats related to dental procedures at more intense emotional and cognitive levels [7,42]. This may be related to greater attention to health-related issues among women or to sociocultural differences in emotional expression [43]. The higher pain and dental anxiety levels observed among individuals with higher income may be associated with differences in treatment-related expectations [44]. In addition, elevated anxiety levels among unemployed individuals may reflect associations between socioeconomic circumstances and anxiety-related perceptions. The low prevalence of traumatic dental experiences in the present sample may suggest that psychosocial and cognitive factors, in addition to previous trauma, could be relevant to dental anxiety–fear [2].

Regarding endodontic variables, differences according to tooth type and treatment status were more pronounced in state–trait anxiety levels than in dental fear scores. Higher anxiety levels were observed in patients with previously treated anterior teeth and in those requiring retreatment of anterior teeth. Because anterior teeth play an important role in esthetics and social appearance, these findings may be consistent with the possibility that concerns regarding treatment outcomes and perceived consequences are associated with anxiety levels independent of dental fear [45]. Additionally, dental anxiety–fear was not significantly associated with objective endodontic variables including tooth type, treatment status, or radiographic disease severity. This finding aligns with previous evidence suggesting that subjective perceptions are more closely associated with dental anxiety–fear than objective disease-related indicators [46]. 

The significant positive correlation between pulse rate and pain levels agrees with previous studies reporting associations between autonomic responses, pain perception, and emotional state during endodontic procedures [47,48]. Higher state and trait anxiety levels also appeared to be associated with lower oxygen saturation values, potentially reflecting physiological responses related to anxiety. However, the low correlation coefficients suggest that these associations were relatively weak. Therefore, physiological markers alone may provide only limited information regarding patients’ psychological characteristics [44].

The present study evaluated preoperative pain, dental anxiety–fear, and state–trait anxiety within the same endodontic cohort and in an integrated framework. This approach provided an opportunity to examine whether pain may contribute to the observed associations among these psychological variables. However, the cross-sectional design does not permit causal inferences or establish temporal ordering among variables; therefore, the findings require cautious interpretation. The self-report-based scales may be subject to recall or social desirability bias. The absence of detailed social and cultural data, combined with the single-center recruitment design, may limit the generalizability of the findings to different populations and clinical settings. Physiological parameters such as pulse rate and oxygen saturation were included in the study; however, these measurements provide only limited information about the neurobiological mechanisms underlying anxiety and pain. 

The large number of subgroup comparisons performed in this study may have increased the likelihood of chance findings due to multiple testing. In addition, some categories included only a limited number of participants, particularly those reporting traumatic dental experiences, those in the higher income categories, and employed individuals. Therefore, statistically significant findings derived from these small subgroups should be considered exploratory and interpreted with caution until confirmed in future studies with larger and more balanced samples.

Furthermore, the mediation analyses were performed as unadjusted models because the primary aim was to explore the associations among the main psychological variables of interest within the proposed mediation framework rather than to evaluate the independent effects of demographic and clinical characteristics. However, demographic and clinical factors may also be related to anxiety, pain, and dental outcomes. Therefore, the observed association patterns should be considered within the context of potential residual confounding. Future prospective multicenter studies incorporating repeated assessments, additional physiological measures, and fully adjusted analytical models are warranted to enhance generalizability and further clarify the temporal relationships among anxiety, pain, and endodontic treatment outcomes.

## 5. Conclusions

Within the limitations of this cross-sectional study, preoperative pain may contribute to the observed association patterns between dental anxiety and state–trait anxiety. Further prospective studies with larger sample sizes and adjusted analytical models are needed to better understand these associations and determine whether temporal relationships exist among the variables.

## Figures and Tables

**Table 1:** Sociodemographic characteristics of study population.

		*n*	%
Gender	Female	148	58.5
	Male	105	41.5
Educational level	Literate	11	4.3
	Primary School	95	37.5
	High School	109	43.1
	University	38	15.0
Employment status	Unemployed	189	74.7
	Employed	64	25.3
Income level (TRY)	10,000–20,000	71	28.1
	20,001–35,000	120	47.4
	35,001–50,000	56	22.1
	50,001–75,000	3	1.2
	75,001–100,000	3	1.2
Dental visit frequency	Never	2	0.8
	Only when symptomatic	236	93.3
	Regular	15	5.9
Dental visit in last 12 months	No	17	6.7
	Yes	236	93.3
Traumatic dental experience	No	250	98.8
	Yes	3	1.2
Source of dental information	Dentist	248	98.0
	Parent	2	0.8
	Media	3	1.2
Information about procedure	No	39	15.4
	Yes	214	84.6

Descriptive statistics are presented as *n* (%).

**Table 2:** Associations between pain intensity and dental anxiety–fear, and anxiety scales.

		MDAS	DFS	STAI-S	STAI-T
NRS	*r*	0.343	0.153	0.238	0.174
	*p*	<0.001 *	0.015 *	<0.001 *	0.006 *
MDAS	*r*		0.606	0.234	0.214
	*p*		<0.001 *	<0.001 *	0.001 *
DFS	*r*			0.418	0.453
	*p*			<0.001 *	<0.001 *
STAI-S	*r*				0.645
	*p*				<0.001 *

Abbreviations: DFS—Dental Fear Survey, MDAS—Modified Dental Anxiety Scale, NRS—Numerical Rating Scale, STAI-S—State Anxiety subscale of the State–Trait Anxiety Inventory, STAI-T—Trait Anxiety subscale of the State–Trait Anxiety Inventory, and *r*—correlation coefficient. * *p* < 0.05.

**Table 3:** Hayes PROCESS macro-4 analysis of indirect associations involving pain.

Outcome	*β*	S.E.	Lower Bound *β*	Upper Bound *β*	Std. *β*	*t*	*p*	*R* ^2^
STAI-S model								
NRS ← STAI-S	0.0839	0.0254	0.0339	0.1340	0.2039	3.3004	0.0011 *	0.042
MDAS ← STAI-S	0.0920	0.0312	0.0306	0.1534	0.1778	2.9502	0.0035 *	0.129
MDAS ← NRS	0.3502	0.0758	0.2010	0.4994	0.2786	4.6222	<0.001 *	
Mediating role	0.0294	0.0117	0.0100	0.0554				
STAI-T model								
NRS ← STAI-T	0.0781	0.0290	0.0209	0.1353	0.1673	2.6891	0.0076 *	0.028
MDAS ← STAI-T	0.0713	0.0354	0.0015	0.1411	0.1215	2.0123	0.0453 *	0.113
MDAS ← NRS	0.3702	0.0759	0.2207	0.5197	0.2945	4.8765	<0.001 *	
Mediating role	0.0289	0.0126	0.0066	0.0568				

Abbreviations: *β*—Unstandardized regression coefficient, *R*^2^—Explained variance, S.E.—Standard Error, and Std. *β*—Standardized beta coefficient. * *p* < 0.05.

**Table 4:** Comparison of pain, dental anxiety–fear, and state–trait anxiety according to demographic and behavioral characteristics.

		NRS	MDAS	DFS	STAI-S	STAI-T
		Mean ± SD (M)	Mean ± SD (M)	Mean ± SD (M)	Mean ± SD (M)	Mean ± SD (M)
Gender	Female	2.36 ± 3.33 (0)	11.7 ± 4.4 (11)	46.56 ± 15.39 (47.5)	45.46 ± 8.14 (48)	43.66 ± 7.59 (46)
	Male	2.35 ± 3.44 (0)	8.92 ± 3.4 (7)	35.52 ± 13.26 (34)	45.14 ± 8.28 (48)	43.01 ± 6.68 (45)
	Test Statistic	−0.361	−5.367	−5.648	−0.238	0.700
	*p*	0.718	<0.001 *	<0.001 *	0.812	0.484
Educational level	Literate	1.36 ± 2.46 (0)	11.45 ± 6.77 (9)	46.64 ± 15.72 (49)	45.73 ± 8.88 (49)	42.82 ± 7.77 (46)
	Primary School	1.94 ± 3.17 (0)	10.44 ± 4.26 (10)	43.03 ± 16.13 (41)	46.03 ± 8.07 (50)	43.71 ± 6.98 (46)
	High School	2.78 ± 3.63 (0)	10.47 ± 4.04 (10)	40.9 ± 14.35 (39)	44.82 ± 8.63 (48)	43.23 ± 7.6 (45)
	University	2.5 ± 3.22 (0)	10.79 ± 3.97 (11)	41.11 ± 17.15 (37.5)	44.92 ± 7.05 (46.5)	43.21 ± 6.79 (44)
	Test Statistic	3.667	0.743	1.815	1.517	0.108
	*p*	0.300	0.863	0.612	0.678	0.955
Employment	Unemployed	2.4 ± 3.45 (0)	10.91 ± 4.33 (11)	43.51 ± 15.96 (44)	45.42 ± 8.32 (49)	43.7 ± 7.31 (46)
	Employed	2.25 ± 3.16 (0)	9.48 ± 3.78 (9)	37.45 ± 13.15 (37)	45.06 ± 7.84 (48)	42.47 ± 6.92 (44)
	Test Statistic	−0.186	−2.318	−2.511	−0.337	1.179
	*p*	0.853	0.020 *	0.012 *	0.736	0.240
Income level (TRY)	10,000–20,000	1.55 ± 3 (0)	9.46 ± 3.93 (9)	38.63 ± 14.88 (37)	44.86 ± 8.35 (48)	43.13 ± 6.73 (44)
	20,001–35,000	2.64 ± 3.54 (0)	10.78 ± 4.06 (11)	43.19 ± 15.19 (44)	45.87 ± 8.36 (49)	43.33 ± 7.29 (46)
	35,001–50,000	2.77 ± 3.26 (2)	11.64 ± 4.76 (11)	44.36 ± 16.54 (45.5)	44.68 ± 7.95 (47)	43.64 ± 7.98 (45)
	50,001–75,000	0 ± 0 (0)	7.33 ± 2.52 (7)	37 ± 13.53 (38)	49 ± 1.73 (50)	47.67 ± 2.31 (49)
	75,001–100,000	5 ± 5 (5)	9.67 ± 3.51 (10)	33.33 ± 17.39 (27)	43.33 ± 6.66 (40)	43 ± 5.29 (41)
	Test Statistic	11.510	13.085	6.827	1.775	0.305
	*p*	0.021 *	0.011 *	0.145	0.777	0.874
Dental visit frequency	Never	6 ± 5.66 (6)	11.5 ± 0.71 (11.5)	48.5 ± 3.54 (48.5)	43 ± 12.73 (43)	47.5 ± 4.95 (47.5)
	Only when symptomatic	2.31 ± 3.32 (0)	10.51 ± 4.25 (10)	41.53 ± 15.49 (39)	45.31 ± 8.17 (48)	43.26 ± 7.25 (45)
	Regular	2.73 ± 3.81 (0)	11 ± 4.34 (11)	48.27 ± 15.62 (48)	45.87 ± 8.49 (47)	44.87 ± 6.98 (46)
	Test Statistic	2.647	1.022	3.449	0.132	0.675
	*p*	0.266	0.600	0.178	0.936	0.510
Traumatic dental experience	Absent	2.32 ± 3.35 (0)	10.48 ± 4.1 (10)	41.72 ± 15.3 (39.5)	45.28 ± 8.2 (48)	43.33 ± 7.19 (45)
	Present	5.33 ± 4.62 (8)	16.67 ± 10.41 (20)	64 ± 19.92 (75)	49.33 ± 7.23 (53)	48.33 ± 9.5 (48)
	Test Statistic	−1.275	−0.946	−1.954	−1.169	−1.195
	*p*	0.202	0.344	0.051	0.243	0.233
Source of dental information	Dentist	2.33 ± 3.35 (0)	10.52 ± 4.27 (10)	41.73 ± 15.5 (39)	45.26 ± 8.16 (48)	43.29 ± 7.25 (45)
	Parent	3 ± 4.24 (3)	11.5 ± 0.71 (11.5)	52.5 ± 6.36 (52.5)	52 ± 9.9 (52)	48.5 ± 2.12 (48.5)
	Media	4 ± 5.29 (2)	12 ± 1 (12)	56 ± 13.23 (51)	46.33 ± 10.69 (52)	48.33 ± 3.79 (50)
	Test Statistic	0.794	1.984	3.635	1.151	1.232
	*p*	0.672	0.371	0.162	0.562	0.293
Information about procedure	No	2.9 ± 3.87 (0)	12.64 ± 5 (11)	49 ± 18.36 (49)	48.85 ± 7.64 (51)	47.05 ± 6.08 (47)
	Yes	2.26 ± 3.27 (0)	10.17 ± 3.98 (10)	40.7 ± 14.61 (38)	44.69 ± 8.13 (48)	42.72 ± 7.22 (44)
	Test Statistic	−0.694	−2.704	−2.509	−3.161	3.967
	*p*	0.488	0.007 *	0.012 *	0.002 *	<0.001 *

* *p* < 0.05.

**Table 5:** Comparison of pain, dental anxiety–fear, and state–trait anxiety according to endodontic variables.

		MDAS	DFS	STAI-S	STAI-T
		Mean ± SD (M)	Mean ± SD (M)	Mean ± SD (M)	Mean ± SD (M)
Removable prosthesis	Absent	10.61 ± 4.18 (11)	42.58 ± 15.44 (41)	45.54 ± 8.15 (49)	43.58 ± 7.36 (46)
	Present	10.17 ± 4.6 (10)	38.36 ± 15.61 (36)	44.06 ± 8.39 (46)	42.25 ± 6.28 (43)
	Test Statistic	−0.824	−1.105	−1.464	−1.002
	*p*	0.410	0.269	0.143	0.316
Teeth requiring primary endodontic treatment	Anterior	9.61 ± 4.79 (9)	42.13 ± 16.64 (41)	43.61 ± 9.62 (48)	44.22 ± 7.66 (46)
	Posterior	10.62 ± 4.02 (11)	42.24 ± 15.65 (41)	44.89 ± 8.18 (48)	43.04 ± 7.41 (45)
	Anterior and posterior	11.73 ± 5.97 (11)	42.64 ± 18.45 (38)	44.73 ± 6.63 (48)	43.36 ± 7.71 (46)
	Test Statistic	3.671	0.038	0.455	0.469
	*p*	0.160	0.981	0.797	0.791
Previously treated teeth	Anterior	11 ± 5.04 (11)	42.00 ± 14.68 (41)	50.53 ± 5.21 (52)	45.94 ± 6.34 (48)
	Posterior	10.27 ± 4.21 (10)	40.88 ± 15.83 (38)	44.09 ± 8.36 (46)	41.69 ± 7.4 (42.5)
	Anterior and posterior	10.17 ± 3.32 (10)	43.59 ± 14.43 (45.5)	46.02 ± 7.82 (49)	44.07 ± 6.83 (45.5)
	Test Statistic	0.183	1.739	10.071	7.071
	*p*	0.912	0.419	0.007 *	0.029 *
Teeth requiring retreatment	Anterior	12.14 ± 4.75 (11)	43.57 ± 14.86 (40.5)	50.64 ± 5.3 (52)	45.43 ± 6.09 (45)
	Posterior	10.73 ± 4.19 (11)	41.75 ± 15.49 (45)	45.28 ± 8.64 (48)	42.96 ± 7.40 (44)
	Anterior and posterior	8.80 ± 2.66 (8)	37.80 ± 10.03 (38)	47.10 ± 7.75 (49)	46.10 ± 5.17 (47.5)
	Test Statistic	3.096	0.765	6.317	2.308
	*p*	0.213	0.682	0.042 *	0.315
PAI score	1	10.45 ± 4.29 (10)	41.14 ± 15.59 (38)	44.97 ± 8.18 (48)	43.18 ± 7.14 (45)
	2	10.20 ± 4.60 (9)	47.50 ± 14.72 (49)	47.55 ± 6.44 (49.5)	45.75 ± 7.32 (48.5)
	3	10.44 ± 3.93 (11)	43.89 ± 12.32 (49)	45.50 ± 8.01 (48)	42.72 ± 7.63 (47)
	4	10.64 ± 4.07 (11)	42.18 ± 16.80 (41)	44.95 ± 9.08 (47)	43.28 ± 7.48 (44)
	5	12.18 ± 4.69 (12)	38.64 ± 12.27 (36)	47.82 ± 6.01 (50)	43.45 ± 6.36 (46)
	Test Statistic	2.517	4.712	2.167	3.351
	*p*	0.642	0.318	0.705	0.501

* *p* < 0.05.

## Data Availability

Data is contained within the article or Supplementary Material.

## Supplementary Materials

The following supporting information can be downloaded at: https://www.mdpi.com/article/10.3390/jcm15124660/s1, Table S1. Clinical characteristics of the study population; Table S2. Distribution of total scores and reliability analyses of the scales used in the study; Table S3. Hayes’ PROCESS macro-4 analysis of the mediating role of pain in the effect of state anxiety on dental fear; Table S4. Hayes PROCESS macro-4 analysis of the mediating role of pain in the effect of trait anxiety on dental fear; Table S5. Correlations Between Demographic and Clinical Characteristics and Anxiety, Dental Fear, and Pain Levels.

## Abbreviations

The following abbreviations are used in this manuscript:

DFS	Dental Fear Survey
ICC	Intraclass Correlation Coefficient
MDAS	Modified Dental Anxiety Scale
NRS	Numerical Rating Scale
PAI	Periapical Index
STAI-S	State Anxiety subscale of the State–Trait Anxiety Inventory
STAI-T	Trait Anxiety subscale of the State–Trait Anxiety Inventory
