# Supplementary material for: When Pain Shapes Dental Anxiety: A Cross-Sectional Mediation Study in Patients Requiring Endodontic Treatment
Source: J Clin Med. 2026 Jun 16;15(12):4660. doi: 10.3390/jcm15124660 (PMC13302241; doi:10.3390/jcm15124660)
Supplement: Supplementary file 1 [file jcm-15-04660-s001.zip › jcm-4339916-supplementary.pdf]

Supplementary Table S1. Clinical characteristics of the study population

Variable	Category	***n***	%
Dental status	Caries present	185	73.1
	Missing teeth present	176	69.6
	Restorations present	207	81.8
Removable prosthesis	Absent	217	85.8
	Present	36	14.2
Tooth requiring primary endodontic treatment	Anterior	23	11.4
	Posterior	168	83.2
	Anterior & posterior	11	5.4
Previously treated tooth	Anterior	17	9.3
	Posterior	120	65.6
	Anterior & posterior	46	25.1
Tooth requiring retreatment	Anterior	14	13.3
	Posterior	81	77.1
	Anterior & posterior	10	9.5
Number of teeth with periradicular radiolucency	None	178	70.4
	One	54	21.3
	Two	19	7.5
	Four	2	0.8
PAI score	1	143	56.5
	2	20	7.9
	3	18	7.1
	4	61	24.1
	5	11	4.3

Descriptive statistics are presented as *n* (%).

Supplementary Table S2. Distribution of total scores and reliability analyses of the scales used in the study

	Min.-Max.	Mean ± SD (Median)	Cronbach's Alpha
MDAS	5-25	10.55±4.23 (10)	0.881
DFS	20-87	41.98±15.50 (41)	0.973
STAI-S	23-60	45.33±8.18 (48)	0.870
STAI-T	28-58	43.39±7.22 (45)	0.831

Supplementary Table S3. Hayes' PROCESS macro-4 analysis of the mediating role of pain in the effect of state anxiety on dental fear

	β	S.E.	Lower Bound β	Upper Bound β	Std. β	t	p	R^2
NRS ← STAI-S	0.0839	0.0254	0.0339	0.1340	0.2039	3.3004	0.0011*	0.042
DFS ← STAI-S	0.7750	0.1103	0.5578	0.9921	0.4092	7.0289	<0.001*	0.188
DFS ← NRS	0.3809	0.2679	-0.1466	0.9085	0.0828	1.4221	0.1562	
Mediating role	0.0320	0.0299	-0.01854	0.1015				

*$p < 0.05$.

Supplementary Table S4. Hayes PROCESS macro-4 analysis of the mediating role of pain in the effect of trait anxiety on dental fear

	β	S.E.	Lower Bound β	Upper Bound β	Std. β	t	p	R^2
NRS ← STAI-T	0.0781	0.0290	0.0209	0.1353	0.1673	2.6891	0.0076*	0.028
DFS ← STAI-T	0.8705	0.1242	0.6259	1.1150	0.4054	7.0105	<0.001*	0.187
DFS ← NRS	0.4527	0.2661	-0.0713	0.9768	0.0984	1.77014	0.0901	
Mediating role	0.0354	0.0285	-0.0069	0.1028				

*$p < 0.05$.

Supplementary Table S5. Correlations Between Demographic and Clinical Characteristics and Anxiety, Dental Fear, and Pain Levels

		NRS	MDAS	DFS	STAI-S	STAI-T
Age†	r	-0.169	-0.009	-0.035	0.056	-0.042
	p	0.007*	0.884	0.577	0.371	0.510
Waiting time†	r	0.025	0.071	0.002	-0.033	-0.041
	p	0.694	0.263	0.974	0.602	0.518
Caries present	r	0.012	-0.008	-0.025	0.026	0.011
	p	0.811	0.865	0.580	0.572	0.810
Missing teeth present	r	-0.119	-0.052	-0.011	0.057	-0.002
	p	0.017*	0.265	0.809	0.211	0.958
Restorations present	r	-0.034	0.001	-0.031	-0.081	-0.126
	p	0.504	0.976	0.489	0.079	0.006*
DMFT	r	-0.118	-0.074	-0.057	-0.020	-0.084
	p	0.015*	0.105	0.195	0.657	0.058
Teeth requiring primary endodontic treatment	r	0.010	-0.036	-0.021	-0.066	-0.032
	p	0.846	0.476	0.658	0.177	0.511
Previously treated teeth	r	-0.062	-0.035	0.003	0.019	-0.073
	p	0.222	0.460	0.944	0.685	0.118
Teeth requiring retreatment	r	0.030	0.039	-0.009	0.079	0.025
	p	0.585	0.449	0.851	0.115	0.621
Heart Rate†	r	0.156	0.076	0.013	-0.073	-0.042
	p	0.013*	0.228	0.831	0.246	0.506
Oxygen Saturation†	r	0.048	-0.012	-0.037	-0.147	-0.126
	p	0.451	0.853	0.555	0.019*	0.046*

*$p < 0.05$. †Spearman correlation.
